# Lymphoma-derived extracellular vesicles inhibit CAR T cell function

**DOI:** 10.17912/micropub.biology.001646

**Published:** 2025-08-05

**Authors:** Khadiza Siddika, Katie R. Flaherty, Reuben Benjamin, Anna Schurich, Molly S. Cook

**Affiliations:** 1 Department of Infectious Diseases, King's College London, London, England, United Kingdom; 2 King's College London, London, England, United Kingdom; 3 King's College Hospital, London, England, United Kingdom

## Abstract

CD19-targeting CAR T cell therapy has shown remarkable efficacy in the treatment of relapsed/refractory B cell lymphoma. However, a proportion of patients exhibit resistance to treatment. We investigate the impact of lymphoma-derived Extracellular Vesicles (EV) on CD19-targeting CAR T cell function *in vitro* . We demonstrate that lymphoma-EV express B cell markers such as CD19 and CD20, which can be transferred to the CAR T cell membrane. In co-culture experiments, lymphoma-EV suppress the tumour-killing capacity of CAR T cells.

**Figure 1. CD19+ Extracellular Vesicles interact with CD19-targeting CAR T cells inducing an activated phenotype and reduced killing capacity:**
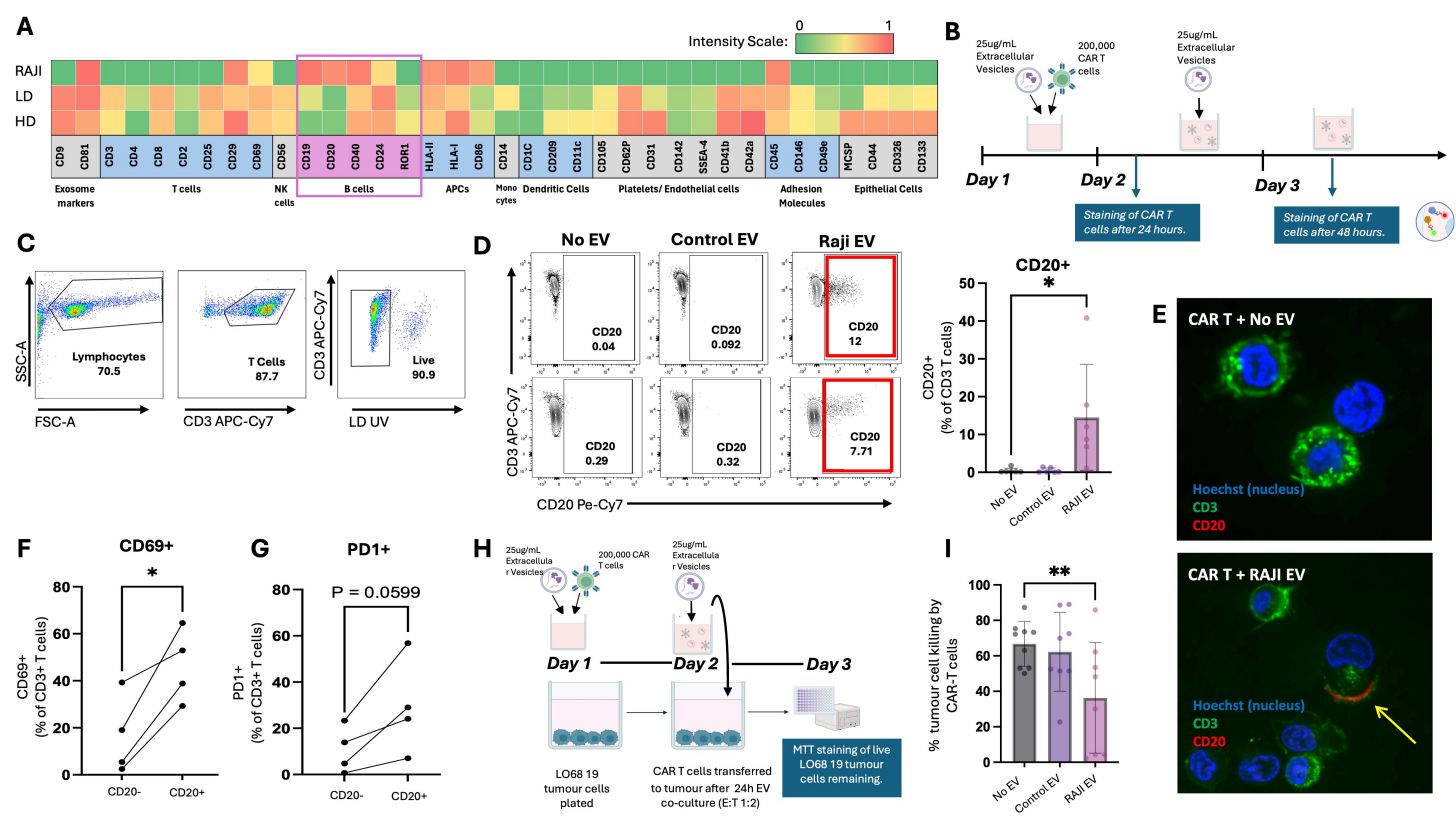
**A:** Heatmap representing the average geometric mean fluorescence intensity signal (GMFI) for 36 standard surface markers on EV derived from RAJI cell culture supernatant (n=1), DLBC lymphoma patient plasma (n=6), and healthy donor plasma (HD, n=10). GMFI values are log normalized between 0 and 1. **B:** Experimental outline for co-culture of CAR T cells with EV. **C:** Gating strategy for live CD3+ T cells. **D:** Flow cytometry plots of live CD3+ T cells against CD20 in two healthy donors after 24-hour co-culture of CAR T cells with vehicle (No EV), Control EV and RAJI EV. Red box highlights CD20+ cells after co-culture. Adjacent graph shows summary data of percent CD20+ cells in the different EV treatment groups (n=6). Statistical significance determined by Wilcoxon test *p<0.05. **E:** Confocal imaging of CAR T cells after co-culture with No EV (top) or Raji EV (bottom) where the T cell marker CD3 (green), B cell marker CD20 (red) and nuclei (blue) are stained. Yellow arrow highlights the interaction of CD20+ EV along CD3+ T cell membrane. **F:** Expression of CD69 in CD20- versus CD20+ CAR T cells following co-culture with RAJI EV for 48 hours (n=4). Statistical significance determined by paired T test , * p<0.05. **G:** Expression of PD1 in CD20- versus CD20+ CAR T cells following co-culture with RAJI EV for 48 hours (n=4). Statistical significance determined by paired T test , * p<0.05. **H:** Experimental outline for CAR T – tumour killing assay using MTT cell staining. **I:** Percent tumour cell killing by CAR T cells, following CAR T cell co-culture with No EV (n=9), Control EV (n=9), and RAJI EV (n=8). Statistical significance shown with Wilcoxon test ** p<0.01.

## Description

Chimeric Antigen Receptor (CAR) T cell therapy involves genetically engineering autologous T cells to express synthetic receptors which enable T cell-directed killing of malignant cells. CAR T cells targeting the B cell marker CD19, is an approved treatment for relapsed/refractory B cell lymphoma. CD19-CAR T cell therapy leads to durable clinical response in ~50% of lymphoma patients *(Boyle et al, 2024) (Neelapu et al, 2017) (Maude et al, 2018) (Schuster et al, 2017)* , the factors underpinning resistance to treatment are not well understood. Here we explore the potential impact of cancer-derived extracellular vesicles (EVs) on CAR-T cell function.

EVs are heterogenous membrane bound vesicles that can be released from all cell types. They are 30-150nm in size, which includes exosomes, microvesicles and apoptotic bodies *(Liu et al, 2021).* The biogenesis of EV involves maturation of early endosomes that are packaged with cell-specific lipids, nucleic acids and proteins. EV membranes are typically enriched in the tetraspanins CD63, CD9 and CD81, along with surface markers from their parental cell. B cell lymphoma cells are known to release EV with tumour-specific protein cargo *(Matthiesien et al, 2022)* and B cell specific surface markers *(Caivano et al, 2015).* In addition, EVs have been shown to play a role in cell-to-cell communication in health and disease *(Chang et al, 2021).* The interaction and transfer of cargo between tumour-derived extracellular vesicles and surrounding cells has been shown to enhance tumour growth, angiogenesis, and extracellular remodelling *(Mashouri et al 2019)* as well as suppress T cell function *(Yamada et al, 2016).*

We isolated EVs from the plasma of lymphoma donors (LD) and healthy donors (HD) and from the supernatant of the B cell lymphoma line, Raji, and assessed EV surface markers using a flow-cytometry based assay. We find that both Raji EV and lymphoma donor EV carry the B cell marker CD19 ( Figure 1A ). However, no EV expressing CD19 were found in healthy donor samples ( Figure 1A ). This could suggest a higher frequency of CD19+ B cells in the peripheral blood of lymphoma donors, although DLBCL donors tend to have similar or lower B cell counts compared to healthy donors ( *Hou et al, 2021 and Geuna et al, 2014* ), it could also suggest that malignant CD19+ B cells produce more EV than healthy CD19+ B cells. In addition, Raji EV carried other B cell markers such as CD20, CD40 and CD24 ( Figure 1A ), making them a suitable model of lymphoma-derived EV.

To explore the potential interaction between CD19+ lymphoma EVs with CD19-targeting CAR T cells, we set up co-culture assays ( Figure 1B ). CAR T cells were co-cultured with 25 µg/mL Raji EV, which is 2.5x the average concentration of EV in the blood ( *Johnsen et al, 2019 and Swerdlov 2012* ), to reflect the enrichment of EV in the tumour microenvironment ( *Belényesi et al, 2025* ). Due to the short half-life of EV ( *Morshita et al, 2017* ), a further 25 µg/mL EV was added to culture after 48h to maintain constant exposure. Flow cytometry was performed at two time points to assess the phenotype of live T cells ( Figure 1C ). Surprisingly, we could detect the B cell marker CD20 on CAR T cells that had been incubated with Raji EV for 24 hours ( Figure 1D ). This suggested interaction between the EV and CAR T cells, potentially via EV-CD19 binding to the anti-CD19 CAR receptor, leading to transfer of CD20 to the CAR T cell membrane. The presence of CD20 alongside the CD3+ CAR T cell membrane was confirmed by confocal imaging ( Figure 1E ). Following this finding, we compared the phenotype of CAR T cells that *had* interacted with EV (CD20+) with those that *had not* interacted with EV (CD20-). We found higher expression of the activation markers CD69 and PD1 on CD20+ versus CD20- CAR T cells ( Figure 1F, G). Overall, this could suggest that CD19+ lymphoma EV can interact with CD19-targeting CAR T cells and induce CAR T activation, potentially via CD19-CAR interaction. Alternatively, it could suggest that phenotypically activated CD69+ PD1+ CAR T cells are more likely to interact with lymphoma EV than other CAR T cell subsets.

The ability of CAR T cells to effectively kill tumour cells is essential to therapeutic outcome in patients. We therefore assessed the impact of lymphoma EV on CAR T cell killing capacity. We set up a cytotoxic assay using CAR T cells that had been co-cultured with Raji EV for 24 hours and plated these onto a CD19+ tumour cell line ( Figure 1H ). The number of live tumour cells was assessed after 24 hours. Having shown that lymphoma EV induced CAR T cell activation, we hypothesised that the CAR T cells would have increased killing capacity. However, we found that following incubation with lymphoma EV, CAR-T cells had significantly reduced tumour-killing capacity in comparison to the control group ( Figure 1I ). This could be due to CD19 on the EV blocking the CAR receptors and preventing interaction with the tumour expressed CD19. Repeat activation induced by the EV could also cause T cell anergy, which has been previously shown *(Zhu et al, 2022),* making them less effective at tumour killing. It will be important in future studies to assess a wider variety of markers on CAR T cells to fully understand the phenotype induced by interaction with lymphoma EV.

## Methods

**Human blood and plasma samples**

Blood samples were obtained from healthy volunteers with approval of the National Health Service Research Ethics Committee (reference 09/H0804/92 and 18/WS/0047), under The Guy’s and St Thomas License (12121). Plasma samples from lymphoma donors were obtained from the King’s College Denmark Hill Haematology Biobank under REC approval NE/18/0141 and the Human Tissue Authority (HTA) License Number 12293. All subjects gave informed written consent for inclusion before participation in the study. The study was conducted in accordance with the Declaration of Helsinki.

Table 1: Demographics of donor plasma including health status, age, and sex.

**Table:** 

	No	Age	Sex
Healthy	10	44.5 (26-63)	4 M
Lymphoma	6	65.8 (57-76) 1 unknown	5 M 1 unknown

**CAR T cell production**

Peripheral blood mononuclear cells (PBMC) were isolated from blood samples using density centrifugation and activated with Transact Beads (Miltenyi Biotec). Activated PBMCs were transduced with retroviruses containing the chimeric antigen receptor scFv-4-1BB-CD3ζ (in house). CAR-T cells were expanded with 100IU/mL IL-2 (Miltenyi Biotech) at 37°c at 5% CO2 for 10 days.

**EV isolation and quantification**

Supernatant was collected from Raji cell lines (ECACC #85011429) grown in serum-free media. EVs were isolated from supernatant using the Total Exosome Isolation Kit (for cell culture media) (Invitrogen). EVs were isolated from cryopreserved plasma from healthy and B cell lymphoma donors using an immuno-bead CD63 human exosome isolation kit (Miltenyi Biotec). EV were also isolated from FCS as a control. Pierce BCA Protein Assay Kit (Thermo Scientific) was used to quantify the protein concentration.

**Multiplex EV assay**

The EVs characterisation was completed using a multiplex bead-based flow cytometry assay to detect EV surface proteins (MACSPlex Exosome Kit, Miltenyi Biotec). The LSR Fortessa 3 flow cytometer and Flow Jo software v9 was used to analyse data.

**CAR T and EV Co-culture Assay**

CAR T cells were plated at a concentration of 1x10 ^6^ cells/ml in cRPMI (RPMI 1640 supplemented with 10% FBS, sodium pyruvate (1mM), L-Glutamine (2mM), non-essential amino acids (0.1mM) and hepes buffer (10mM)). EV samples were added at a final concentration of 25μg/mL. PBS was added as ‘No EV’ condition and FCS EV as ‘control EV’ condition. 25 µg/mL EV was added after a further 24 hours. CAR T cells were analysed at 24 hour and 48-hour timepoints. Cells were stained with live dead-UV (Invitrogen Thermo Fisher Scientific) and surface markers including CD3-APC-Cy-7 (Biolegend), CD20-PE-Cy-7 (Biolegend), CD8-AF700 (Biolegend), PD1-PE (Biolegend), and CD69-BV605 (Biolegend) for 30mins at 4°C in the dark. The cells were acquired on an LSR Fortessa 3 flow cytometer and data analysed using Flow Jo v9.

**Confocal Imaging**

CAR T cells and EV were co-cultured for 24h, then CAR T cells were stained with CD3-FITC (Biolegend), CD20-Alexa-Fluor-700 (Biolegend) and Hoescht dye (ThermoFisher) for 45 minutes at 37ºc. Imaging and analysis were carried out using a 60x water immersion confocal microscope (SoRa, Nikon) for image acquisition and NIS Elements Software (Nikon) and ImageJ (Fiji) software for analysis.

**CAR T tumour killing assay**

The target tumour cells, LO68 (ECACC #10092311) modified to express CD19, were seeded and left for 18 hours to adhere and proliferate. After 24 hours of EV co-culture, CAR T cells were plated on the target tumour cells at a CAR T : tumour cell ratio of 1:2 and incubated for 18 hours at 37°C. CAR T cells were removed and remaining tumour cells were stained with 3-(4,5-dimethylthiazol-2-yl)-2,5-diphenyl-2H-tetrazolium bromide (MTT) at 5mg/ml in cRPMI and incubated at 37°C for 1 hour. MTT was aspirated and DMSO (Sigma Life Science) was added. The absorbency was measured on the MPM Absorbance 96 plate reader and percent tumour cells killed by CAR T cells was calculated as 100 - ((absorbance of co-culture well / absorbance of tumour alone) * 100).

**Statistical analysis**

All statistical analysis was run on Prism 9 (Graphpad). A one-way ANOVA multiple comparisons test was used to compare impact of the multiple different EV groups. For data normalised to the PBS control, a Wilcoxon Test was used to compare EV groups with the control value of 1. For paired data, the difference between the two groups was analysed by paired T test.
